# Impact of Ultrasound Pretreatment and Enzyme Concentration on Taste and Biological Activities of Porcine Lung Hydrolyzates

**DOI:** 10.3390/foods14183243

**Published:** 2025-09-18

**Authors:** Manuel Ignacio López-Martínez, Fidel Toldrá, Sandra Morcillo-Martínez, Leticia Mora

**Affiliations:** Instituto de Agroquímica y Tecnología de Alimentos (CSIC), Avenue Agustín Escardino 7, 46980 Paterna, Valencia, Spain; mi.lopez@iata.csic.es (M.I.L.-M.); ftoldra@iata.csic.es (F.T.); sandramorcillo.m@hotmail.es (S.M.-M.)

**Keywords:** porcine lung, enzyme concentration, ultrasound, taste, biological activities, in silico approaches

## Abstract

The revalorization of porcine meat by-products is necessary to reduce their environmental and economic impact. Porcine lungs are usually discarded or used for low-value purposes despite their richness in collagen, elastin, or phospholipids. Enzymatic hydrolysis, in combination with ultrasound pretreatment, improves the generation of hydrolyzates with biological and taste-enhancing properties. The main objective of this study was to evaluate the impact of ultrasound pretreatment and enzyme concentration in the development of functional and taste-rich porcine lung hydrolyzates. Ultrasound pretreatments significantly increased the degree of hydrolysis and the antioxidant activity in 1:100 Enzyme: Substrate (E/S) ratio hydrolyzates. On the other hand, the combination of 1:20 E/S concentration with ultrasound pretreatment significantly increased the umami free amino acids content and Equivalent Umami Concentration (EUC), being the last one comparable with other umami-rich foods and ingredients. In silico predictions showed that the use of ultrasound pretreatment enhances the percentage of potential bioactive peptides according to PeptideRanker, whereas the bioinformatics tools UMPred-FRL and BERT4Bitter showed more umami peptides than bitter in all the hydrolyzates. These results suggest the combination of ultrasound pretreatment with 1:20 E/S can be a good strategy to revalorize porcine lung by producing hydrolyzates that could be used as a functional ingredient.

## 1. Introduction

Recent population growth has increased the demand for high-biological-value proteins—those with high digestibility and a complete profile of essential amino acids—driving improvements in meat industry production. Thus, around 345 tonnes of meat are produced worldwide, from which 155 tonnes of by-products such as skin, feathers, bones, or organs are obtained. In order not avoid their environmental and economic impact, by-products must be revalued in an appropriate manner [1]. A percentage of 55% of pig slaughterhouse by-products would represent edible by-products also called co-products [2], among which organs, such as lungs, stand out due to their excellent nutritional value rich in proteins of high biological value, vitamins, and minerals [3]. Due to its high protein content, enzymatic hydrolysis can be a very interesting strategy to revalue porcine organs, as it favours the development of hydrolyzates rich in bioactive peptides, which can exert numerous biological functions, such as antihypertensive, antidiabetic, or antioxidant [4]. Moreover, it has been investigated that the use of certain enzymes such as Flavourzyme improves the perception of meat taste through the release of certain peptides and free amino acids [5].

Despite its advantages, the industrial scale-up of enzymatic hydrolysis is a difficult and expensive process [6]. Pretreatment techniques such as ultrasound favour the unfolding of the protein structure releasing new cleavage sites for the enzyme, which could lead to the development of more efficient hydrolyzates, potentially reducing the time, amount of enzyme used, and making the process cheaper [7]. Moreover, the use of advanced in silico tools provide valuable insights into the peptidomic profile of hydrolyzates, enabling the prediction of their gastrointestinal behaviour, absorption bioactivity, and potential taste attributes [8].

The main objective of this study was to evaluate the impact of ultrasound pretreatment and enzyme concentration and on the preparation of porcine lung hydrolyzates with enhanced taste and antioxidant properties. The degree of hydrolysis and antioxidant activities were analyzed using in vitro assays, and free amino acids content and total nucleotide content were determined using high-performance liquid chromatography (HPLC). Furthermore, the best hydrolyzates were subjected to peptidomic analysis by mass spectrometry in tandem, followed by in silico predictions to evaluate their potential bioactivity and taste profiles.

## 2. Materials and Methods

### 2.1. Chemicals and Reagents

Flavourzyme 1000L was purchased from Novozymes (Bagsværd, Denmark). All high-performance liquid chromatography (HPLC) reagents, including acetonitrile (ACN), methanol (MeOH), and ethanol (EtOH), were purchased from Scharlau (Barcelona, Spain) in analytical grade. Potassium ferricyanide, potassium persulfate, sodium hydroxide, ferric chloride, trichloroacetic acid (TCA), trifluoroacetic acid (TFA), formic acid (FA), butylated hydroxytoluene (BHT), 2,2′-azino-bis(3-ethylbenzothiazoline-6-sulfonic acid) (ABTS), 2,2-diphenyl-1-picrylhydrazyl-hydrate (DPPH), o-phthalaldehyde (OPA), sodium dodecyl sulphate (SDS), DL-dithiothreitol (DTT), iron (II) chloride tetrahydrate, sodium 4-[3-(pyridin-2-yl)-6-(4-sulfophenyl)-1,2,4-triazin-5-yl]benzene-1-sulfonate (Ferrozine), 6-hydroxy-2,5,7,8-tetramethylchroman-2-carboxylic acid (Trolox), fluorescein, 2,2′-azobis (2-methylpropionamidine) dihydrochloride (AAPH), perchloric acid, amino acids, and nucleotides standards were obtained from Sigma (St. Louis, MO, USA). Sodium acetate, di-sodium tetraborate decahydrate, monobasic sodium phosphate, potassium dihydrogen orthophosphate, potassium hydroxide, ethylenediaminetetraacetic acid (EDTA), and ascorbic acid were purchased by PanReac (Darmstadt, Germany).

### 2.2. Proximate Composition and Physicochemical Properties

#### 2.2.1. Sample Preparation

Three different fresh porcine lungs were provided by a local slaughterhouse and immediately weighed and cut into small pieces. The samples were then subjected to heat treatment at 90 °C for 10 min in a thermostatic water bath and cooled in an ice bath. Later, cooked porcine lungs were grounded using a meat mincer (Moulinex, Alençon, France), vacuum-packaged, and stored at −20 °C until further analysis.

#### 2.2.2. Physicochemical Properties and Proximate Composition

Water activity (a_w_) was measured with an a_w_ metre (Aqualab^®^ 4Tev, Meter Food, Pullman, WA, USA), while pH was determined using a meat pH metre (HI 99163, HANNA Instruments, Woonsocket, RI, USA) at 25 °C. A Minolta handheld colorimeter (CR-400, Konica Minolta, Tokyo, Japan), calibrated with a white standard calibration plate (L* = 97.43, a* = 4.38, b* = 4.09), was used to obtain the colour parameters (luminosity (L*), redness (a*), and yellowness (b*)). Hue and saturation (Chroma) were calculated using the following equations:

$$Hue\;\left(h{}^{\circ}\right)=\arctan\;(\frac{b\;*}{a\;*})$$

$$Chroma\;\left(C\;*\right)=\sqrt{a^{*2}+b^{*2}}$$

All measurements were performed in triplicate.

Moisture content was determined using a thermogravimetric balance (HB43 Halogen, Mettler Toledo, Columbus, OH, USA) according to the manufacturer’s instructions. Protein content was analyzed using Dumas combustion method according to the Association of Official Agricultural Chemists (AOAC) method 992.15 [9]. All measurements were performed in triplicate. The results for all parameters were expressed in percentage and reported as mean values.

### 2.3. Enzymatic Hydrolysis Procedure

#### 2.3.1. Sample Preparation

250,000 mg of minced cooked porcine lung was diluted with 500 mL of double-distilled water and homogenized at 4 °C for 5 min using a masticator homogenizer (IUL Masticator Classic Panoramic, 400 mL, Microplanet, Barcelona, Spain). The mixture was grounded with a polytron (Polytron™ 2500E, Kinematica, Malters, Switzerland) at 18,000 rpm for 3 min in ice bath.

#### 2.3.2. Ultrasound Pretreatment

Prior to enzymatic hydrolysis, 250 mL of 500 mg/mL dilution was subjected to ultrasound pretreatment using an ultrasonic probe (Sonics Materials VCX-750-220, Thermo Fisher, Waltham, MA, USA) with the following conditions: Frequency of 20 kHz, power density of 750 W/mL, and amplitude of 75% during 90 min with 2 s of pulses on and 4 s of pulses off.

#### 2.3.3. Enzymatic Hydrolysis

Different concentrations (1:100, 1:40 and 1:20 Enzyme:Substrate ratio (*w*:*w*) (E/S)) of Flavourzyme 1000L were tested. The hydrolysis procedure was performed in 500 mg/mL porcine lung dilutions during 120 min at 50 °C with agitation of 500 rpm.

All hydrolyzates were carried out in triplicate and subjected to thermal treatment at 85 °C for 10 min to inactivate the enzyme. Samples were cooled in an ice bath and stored at −20 °C until use.

### 2.4. Degree of Hydrolysis

The degree of hydrolysis (DH) was assessed following the method of Nielsen et al. (2001) [10]. A 36 μL aliquot of 5 mg/ mL diluted sample was mixed with 270 μL of OPA reagent and incubated at 25 °C for 2 min. Absorbance was measured at 340 nm using a plate reader (CLARIOstar^®^ Plus, BMG Labtech, Ortenberg, Germany). DH was calculated with the following formula using meat protein parameters (α = 1.00; β = 0.40; h_tot_ = 7.6):

Degree of hydrolysis (DH) = h/h_tot_ × 100%

where h represents the whole content of hydrolyzed peptide bonds, and h_tot_ corresponds to the total peptide links per protein equivalent. All assays were performed in triplicate.

### 2.5. Determination of Taste-Related Substances

#### 2.5.1. Free Amino Acids (FAA) Determination

250 μL of 5 mg/mL hydrolyzate dilution was mixed with 40 μL of the internal standard (5 mmol/L norleucine) and derivatized following the Pico-tag method of Aristoy & Toldrá (1991) [11]. Chromatographic separation was carried out on an HPLC system (Acquity Arc CH/CHC Core Fluidics; Waters, C USA) using a reverse-phase Waters Pico Tag^®^ C18 column (60 Å, 4 µm, 3.9 mm × 300 mm; Waters Corp., Milford, MA, USA) as described by Flores et al. (1997) [12]. The mobile phases consisted of ACN:H_2_O:MeOH (45:40:15) and 70 mmol/L sodium acetate buffer with 2.5% ACN (*v*:*v*) (pH 6.55). Analysis conditions included a column temperature of 52 °C, sample temperature of 12 °C, flow rate of 1 mL/min, and detection wavelength at 254 nm. Free amino acids were quantified using standard curves, and results were expressed as mg FAA/g wet matter. All assays were performed in triplicate, and mean values were reported. Taste attributes of amino acids were sourced from previous publications [13,14,15,16].

#### 2.5.2. Nucleotides Determination

The extraction of total nucleotides was performed following the method of Burns & Ke (1985) [17]. A total of 4 g of minced lungs were homogenized with 15 mL of 0.6 mol/ L HClO_4_ using a homogenizer (IUL Masticator Classic Panoramic, 400 mL, Microplanet, Spain) for 4 min at 4 °C. The homogenate was centrifuged at 24,500× *g* for 20 min at 4 °C, and the resulting supernatant was filtered through glass wool at 25 °C. Samples were introduced into an ice bath, and their pH was adjusted to 6.5 by adding 3 mol/L K_2_CO_3_. Subsequently, they were centrifuged at 24,500× *g* for 10 min, and supernatants were mixed with ACN (1:1) (*v*:*v*) and filtered through a 0.22 μm nylon membrane. Total nucleotide analysis was conducted according to Mora et al. (2010) method [18] using an HPLC system (Acquity Arc CH/CHC Core Fluidics; Waters, Milford, MA, USA) and HILIC separation with a ZIC^®^-pHILIC column (4.6 × 150 mm, 5 μm) and a guard column (2.1 × 20 mm, 5 μm). The mobile phases were four, two ammonium acetate buffers (150 mmol/L, pH 3.5 and 100 mmol/L, pH 7), ACN, and double-distilled water. Chromatographic conditions were set at a column temperature of 28 °C, sample temperature of 12 °C, flow rate of 0.5 mL/min, and detection wavelength at 254 nm. Nucleotide quantification was performed using standard curves, and results were expressed as mg nucleotides/100 g of wet matter. All analyses were conducted in triplicate, and mean values were reported. Nucleotides theorical tastes were compiled from previously published data [13,15,16,19,20].

#### 2.5.3. Equivalent Umami Concentration (EUC)

The equivalent umami concentration (EUC) is a parameter that quantifies the relative intensity of umami taste in food products by evaluating the synergistic effect between umami free amino acids and nucleotides [21]. EUC is calculated with the following formula:

EUC (g MSG/100 g wet matter) =Ʃa_i_b_i_ + 1218Ʃa_i_b_i_ (Ʃa_j_b_j_)

where a_i_ and aⱼ is the content of umami free amino acids, aspartic acid (Asp) and glutamic acid (Glu), and umami-related nucleotides inosine monophosphate (IMP), guanosine monophosphate (GMP), and adenosine monophosphate (AMP), respectively. The terms b_i_ and bⱼ reported their relative umami contributions (RUC) in comparison to monosodium glutamate (MSG) (Asp = 0.077, Glu = 1) for free amino acids and to inosine monophosphate (IMP) (IMP = 1, GMP = 2.3, AMP = 0.18) for nucleotides. The constant 1218 accounts the increment due to the synergy between umami amino acids and nucleotides. EUC values, obtained from triplicate measurements, were expressed as g MSG/100 g wet matter.

#### 2.5.4. Taste Active Value (TAV)

The taste activity value (TAV) quantifies the contribution of taste-related substances to the overall taste profile of a food product [13]. It is calculated as the ratio between the concentration of a given compound (C) and its taste threshold (T):

$$Taste\;activty\;value\;\left(TAV\right)=\frac{C}{T}$$

TAV values above 1 indicate that the compound has a perceptible effect on taste, with higher values signifying a greater contribution. TAV values are dimensionless and were determined in triplicate. Taste thresholds for amino acids and nucleotides were obtained from published data [13,14,15,22,23].

### 2.6. Biological Activity Assays

#### 2.6.1. Sample Preparation

In total, 0.2 mg/mL hydrolyzates was used for the Oxygen Radical Antioxidant Capacity (ORAC) assay. The assays of DPPH and ferric reducing antioxidant power (FRAP) and Fe^2+^ ion chelating power were carried out using deproteinized lung samples by adding 300 μL of EtOH to 100 μL of hydrolyzates at 500 mg/mL concentration and incubated for 960 min [24]. Subsequently, they were centrifuged at 20,879× *g* for 5 min at 4 °C. Supernatants were collected and stored at −20 °C until use. A 1:4 (*v*:*v*) dilution of the deproteinized sample with EtOH was used in ABTS assays.

#### 2.6.2. Antioxidant Activity Assays

In vitro antioxidant activity was evaluated using five different methods: (i) DPPH radical-scavenging assay, following the method of Bersuder et al. (1998) [25], reported results as percentage of inhibition; (ii) ABTS assay was conducted according to Re et al. (1999) [26], with results expressed as mmol of Trolox equivalent antioxidant capacity (TEAC) per mg of sample; (iii) FRAP assay were performed based on Chen et al. (2010) methodology [27], and results were expressed as 700 nm Absorbance Units (AU); (iv) ORAC assay was carried out according to Davalos et al. (2004) [28], obtaining results in mmol of Trolox equivalent per gram of wet matter; and (v) Fe^2+^ ion chelating capacity following the Zheng et al. (2019) method [29], with results expressed as percentage of chelation. All assays were assessed in triplicate.

### 2.7. MS/MS Analysis

#### 2.7.1. Sample Preparation

In total, 500 mg/mL porcine lung hydrolyzate samples were deproteinized by adding 3 volumes of EtOH and incubated for 960 min following the Mora et al. (2015) protocol [24]. A total of 150 μL of the deproteinised sample was vacuum-dried (SpeedvacSPD120, Thermofisher, Waltham, MA, USA) at 35 °C for 240 min. Then, the samples were resuspended in 20 μL of double-distilled water: MeOH 50:50 (*v*:*v*) with 0.1% of TFA.

#### 2.7.2. Tandem Mass Spectrometry Analysis

A total of 0.5 µL of deproteinized sample was further diluted to 20 μL with 0.1% FA and loaded in an Evotip pure tip (EV2018, EvoSep, Odense, Denmark) according to the manufacturer’s instructions. Tandem mass spectrometry analysis (LC–MS/MS) was performed in a Tims TOF fleX mass spectrometer (Bruker, Billerica, MA, USA). The sample loaded in the Evotip pure was eluted on an analytical column PepSep (10 cm × 150 µm, 1.5 µm; Evosep, Denmark) using the Evosep One platform and resolved with the 30 SPD chromatographic method defined by the manufacturer. The eluted peptides were ionized in a captive spray with 1700 V at 200 °C and analyzed in a ddaPASEF mode with the following TIMS settings: custom mode, 1/K0: 0.7–1.76 V.s/cm^2^, ramp time: 100 ms, Duty Cycle 100%, and Ramp rate: 9.42 Hz. The used MS settings have been scanned from 100 to 1700 *m*/*z* in scan mode PASEF and positive ion polarity. The used MSMS settings have been a total of 4 PASEF ramps, a total cycle time of 0.5 s, minimum charge of 0, and maximum charge of 5. The scheduling was targeting intensity of 12,500 and intensity threshold of 1000, with active exclusion ON. To include mono charged peptides, the inclusion polygon was deleted, allowing the selection of precursors at the 1/K0 completed space (IMS). The system sensitivity was controlled with 20 ng of HELA digested proteins. For protein identification, MSFragger searches were performed (via FragPipe) for the identification of non-tryptic peptides. Database was generated for Uniprot_*Sus Scrofa*. A total of 4646 proteins were identified using the 30 SPD gradient.

### 2.8. In Silico Analysis

The potential bioactivity of peptides were predicted with PeptideRanker [30] (http://distilldeep.ucd.ie/PeptideRanker/, accessed on 19 July 2025) in which, values above 0.5 are considered potentially bioactive. However, only peptides with values higher than 0.9 were considered for further in silico analysis. For the prediction of antioxidant potential, samples were analyzed using AnOxPePred 1.0 (https://services.healthtech.dtu.dk/services/AnOxPePred-1.0/, accessed on 19 July 2025) [31]. The dipeptidyl peptidase IV inhibitory potential of the identified peptides was studied using StackDPPIV [32] (https://pmlabstack.pythonanywhere.com/StackDPPIV, accessed on 19 July 2025) and Angiotensin Converting Enzyme (ACE) inhibitory potential using Deepstack-ACE [33] (https://pmlabqsar.pythonanywhere.com/predict_DeepstackACE, accessed on 19 July 2025). To assess the safety of peptides, ToxinPred tool [34] (https://webs.iiitd.edu.in/raghava/toxinpred/ accessed on 19 July 2025) was used to predict potential toxicity of peptides and their physicochemical properties. Allergenicity was evaluated using AllerTOP v.2.0 [35] (https://ddg-pharmfac.net/allertop_test/, accessed on 19 July 2025), cell-permeating capacity was analyzed using MLCPP [36] (http://www.thegleelab.org/MLCPP/MLCPP.html, accessed on 19 July 2025), and antimicrobial potential was assessed with DBAASP [37,38] (https://dbaasp.org/tools?page=linear-amp-prediction, accessed on 19 July 2025). In silico gastrointestinal digestion of >0.9 PeptideRanker sequences was performed using BIOPEP-UWM tool [39,40] (https://biochemia.uwm.edu.pl/biopep-uwm/, accessed on 19 July 2025) by choosing pepsin (pH 1.3, EC 3.4 23 1), trypsin (EC 3.4 21 4), and chymotrypsin C (EC 3.4 21 2) as digestive proteases. The fragments obtained from this process were compiled and compared with the BIOPEP-UWM library to determine previously reported biological activities or taste. Regarding the prediction of taste potential, UMPred-FRL [41] (https://pmlabstack.pythonanywhere.com/UMPred-FRL, accessed on 19 July 2025) and BERT4Bitter (https://pmlab.pythonanywhere.com/BERT4Bitter) [42] (accessed on 19 July 2025) tools were used to evaluate the potential umami and bitter taste of the identified sequences, respectively.

### 2.9. Statistical Analysis

Statistical analysis of the data was carried out by one-way analysis of variance (ANOVA), followed by Tukey’s rank test, with a significance threshold of *p* < 0.05. Different lowercase letters (a, b, c, d, e, f) indicate significant differences among all samples. Different uppercase letters (A, B, C, D) denote significant differences within the group of samples without ultrasound pretreatment. Different uppercase letters (E, F, G, H) denote significant differences within the group of samples with ultrasound pretreatment. Different uppercase letters (X, Y) indicate significant differences between pairs of samples with the same E/S ratio (0, 1/100, 1/40, 1/20). Pearson correlation analyses were also performed on the biological activities and taste-related substances concentration with the degree of hydrolysis. Minitab version 21 (Minitab, LLC, State College, PA, USA) was the statistical software used for this analysis.

## 3. Results

### 3.1. Porcine Lung Characterization

According to Table 1, the values obtained for moisture, protein, pH, and water activity are comparable to those reported in the literature by other authors [43,44,45].

Cooked porcine lungs exhibited low chroma values, which means that their overall colour was not saturated. Hue angle corresponded to reddish colours (0–90). The total nucleotide content is consistent with values reported in a previous study in porcine organs [44]. The concentration of bitter nucleotides (inosine and hypoxanthine) (46.5 mg/100 g wet matter) is almost ten times higher than that of umami nucleotides (AMP, IMP, and GMP) (4.64 mg/100 g wet matter). Also, the taste activity value (TAV) analysis reveals that none of the umami nucleotides exceed a TAV of 1, indicating that they do not have a noticeable impact on the overall taste of cooked porcine lungs. Nevertheless, it is reported that umami 5’-nucleotides can exhibit a synergistic effect when combined with certain amino acids, such as aspartic acid (Asp (D)) and glutamic acid (Glu (E)), enhancing the umami taste [13]. Thus, although nucleotides might have no impact on taste by themselves, they could influence the final umami taste when combined with other amino acids generated after hydrolysis.

### 3.2. Degree of Hydrolysis and Taste-Related Substances Analysis

According to the results shown in Table 2, the degree of hydrolysis (DH) is mostly influenced by enzyme concentration, with a significant increase (*p* < 0.05) with higher amounts of enzyme. Pearson correlation analysis supports this theory, showing a strong positive correlation near to 0.9 (ρ = 0.89) between enzyme concentration and DH. Ultrasound (US) pretreatment also exerts a positive effect in the increase in DH but is only significant (*p* < 0.05) at 1:100 E/S ratio. The Pearson correlation between US and DH is positive but low (ρ = 0.12), suggesting that US pretreatment may have a positive impact on DH, although to a lesser extent than enzyme concentration. Regarding the free amino acids content (FAA) of porcine lung hydrolyzates, a similar trend was observed, with significant increases in every taste-related (umami, sweet, bittersweet, and bitter) FAA content (*p* < 0.05) as enzyme concentration increase. In fact, a strong positive Pearson correlation has been observed between enzyme concentration and the content of each taste-related FAA (umami: ρ = 0.91; sweet: ρ = 0.96; bittersweet: ρ = 0.96; and bitter: ρ = 0.96) and between DH and each FAA content (Umami: ρ = 0.78; sweet: ρ = 0.84; bittersweet: ρ = 0.85; and bitter: ρ = 0.86). Analyzing the US pretreatment effect, a significant increase (*p* < 0.05) in umami FAA content (10.45 ± 1.81b mg aas/g wet matter < 13.87 ± 2.30a mg aas/g wet matter) and in EUC (47.19 ± 8.93b g MSG/100 g wet matter < 62.85 ± 10.41a g MSG/100 g wet matter) was observed at 1:20 E/S ratio if all samples were analyzed together. If samples were evaluated in pairs with the same E/S substrate, there are significant increases (*p* < 0.05) in umami, sweet, bitter FAA content, and EUC at all E/S ratios. Pearson correlation could support this finding, since its value between US pretreatment and umami FAA content and EUC (Umami: ρ = 0.21; EUC: ρ = 0.22) is higher than the other taste-related FAA contents (Sweet: ρ = 0.14; bittersweet: ρ = 0.13; and bitter: ρ = 0.14). Bitter is the most predominant taste in FAA content, although the total content of pleasant-tasting amino acids (sweet, bittersweet, and umami) at 1:20 E/S ratio is almost double than bitter ones, with no effect of US pretreatment, suggesting an overall pleasant taste potential. These findings can be supported by Taste Activity Value (TAV) analysis (Table 3). At 1:20 E/S ratio with US pretreatment, the influence of Glu (E) (umami), Arg (R), and Lys (K) (bittersweet) increases notably from >15 to >20 for Glu and Lys, and from >10 to >15 for Arg. These results suggest that these hydrolyzates may exhibit an overall taste profile strongly influenced by umami and bittersweet, reinforcing the theory that they can present a potentially pleasant taste.

In protein hydrolyzates, umami, sweet, and bittersweet are considered pleasant tastes, while bitter is considered an undesirable taste [46]. The presence of bitter taste is one of the main disadvantages in protein hydrolyzates. This bitterness is usually related to the release of hydrophobic peptides and certain free amino acids, such as Leu (L) or Tyr (Y) [47]. Therefore, finding strategies for debittering these hydrolyzates can be useful for their potential introduction as an ingredient in a food product. Flavourzyme is a commercial mixture of exo- and endopeptidases with medium hydrolytic capacity. In combination with US, which can induce protein unfolding and facilitate enzyme accessibility, Flavourzyme has proven to be effective in producing hydrolyzates with meaty flavours and desirable tastes, possibly due to the removal of hydrophobic ends and the release of pleasant taste free amino acids [5]. In this sense, a similar effect was observed in a previous study of porcine liver hydrolyzates obtaining umami and bittersweet amino acids [48]. Umami compounds can also modify the overall taste profile of a food product by enhancing some tastes like sweet or salty, and by masking unpleasant tastes like bitter [49]. Several authors have reported that US pretreatment could promote the release of umami-related substances, such as peptides, amino acids, and nucleotides, in protein hydrolyzates obtained from meat and fishery industry by-products [50,51].

These findings are in line with the results of this study since US pretreatment enhances the umami amino acid content and EUC. In fact, the EUC values obtained in this study at 1:20 E/S ratio are comparable to umami-rich foods such as mushroom broths (65.23 ± 0.49 g MSG/100 g) [52] or soy sauce (46.08 – 238.11 g MSG/100 g) [53]. Furthermore, Zhu et al. (2022) reported that EUC can be a reliable parameter for evaluating umami intensity in liquid meat products as EUC correlates with human sensory analysis (ρ = 1.00) [54]. On the other hand, it was recently reported that several basic amino acids, such as Lys or Arg, could enhance the salty perception of foods, allowing the production of salt-reduced products without losing palatability [55]. In this study, the hydrolyzates prepared at 1:20 E/S ratio and US pretreatment could be a good candidate as taste enhancer due to the TAV of these two amino acids showing a significant increase (*p* < 0.05) in EUC and umami amino acid content.

### 3.3. Biological Activity Assays

**Figure 1:**
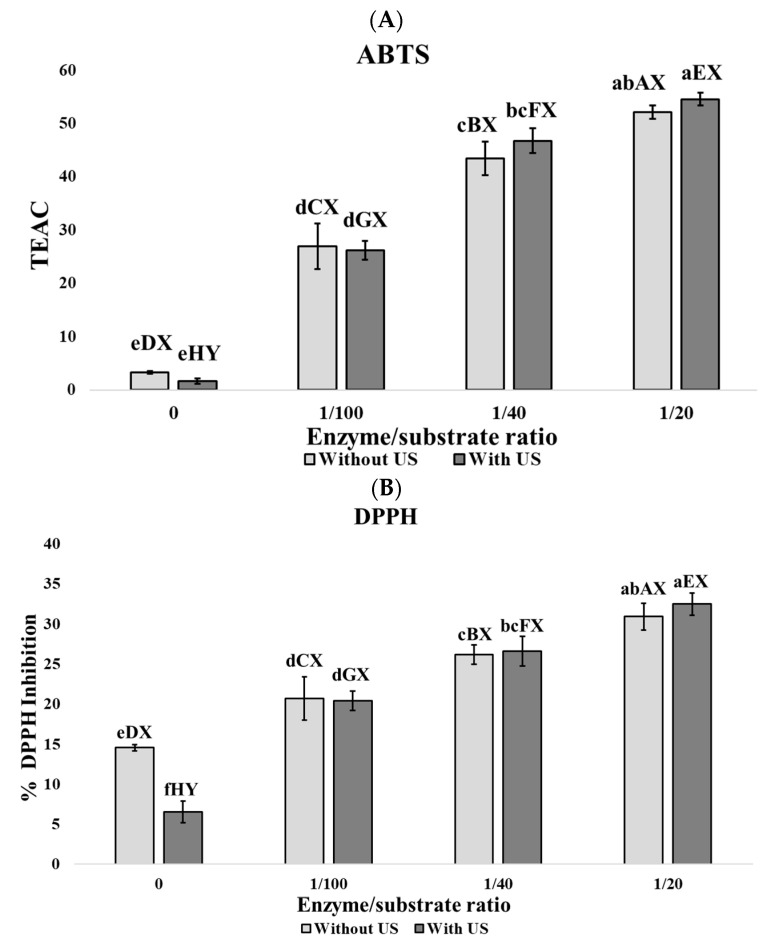

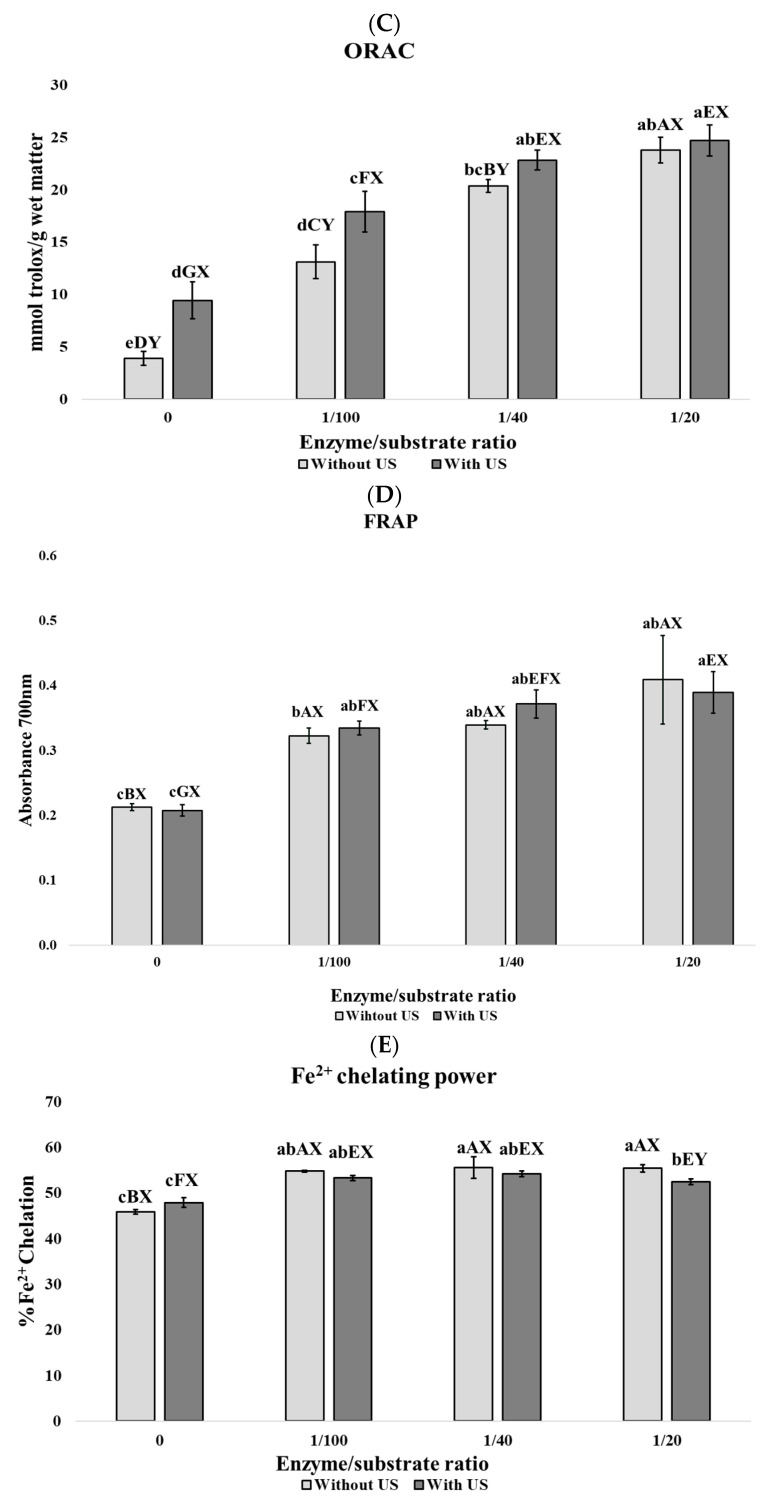
Biological activities of porcine lung hidrolyzates. (**A**) ABTS assay expressed in TEAC values, (**B**) DPPH assay expressed in % of DPPH inhibition, (**C**) ORAC assay expressed in mmol Trolox/g wet matter, (**D**) FRAP assay expressed in Absorbance units at 700 nm, and (**E**) Ferrous Ion chelating activity power expressed as % of chelation. Results are expressed by mean ± SEM of triplicates. Different lowercase letters (a, b, c, d, e, f) denote significant differences among all samples. Different uppercase letters (A, B, C, D) denote significant differences within the group of samples without ultrasound pretreatment. Different uppercase letters (E, F, G, H) denote significant differences within the group of samples with ultrasound pretreatment. Different uppercase letters (X, Y) indicate significant differences between pairs of samples with the same enzyme: substrate ratio (0, 1/100, 1/40, 1/20).

According to Figure 1, enzyme concentration is the most relevant parameter in the increase in antioxidant activities ABTS (Figure 1A), DPPH (Figure 1B), and ORAC (Figure 1C), although not in FRAP (Figure 1D) and Fe^2+^ chelating power (Figure 1E).

In addition, US pretreatment contributed to the enhancement of these activities when all samples were evaluated together, showing a significant increase (*p* < 0.05) in ORAC values at the 1:100 E/S ratio. No significant differences (*p* > 0.05) were observed between the results obtained at the 1:40 E/S ratio with US pretreatment and the 1:20 E/S ratio without US pretreatment in the ABTS, DPPH, and ORAC assays. When samples were evaluated in pairs with the same enzyme-to-substrate ratio, significant differences (*p* < 0.05) in ORAC values were observed at E/S ratios of 0, 1:100, and 1:40. Then, despite enzyme concentration being the most relevant parameter influencing antioxidant activity, US pretreatment positively contributes to the increase in antioxidant activity. In animal protein hydrolyzates, antioxidant activity is usually related to the action of low molecular weight bioactive peptides [56]; therefore, an increase in the degree of hydrolysis is usually related with the generation of small peptides that enhances the antioxidant activity of the hydrolyzates. In this sense, Pearson correlation among antioxidant activities and DH gives values higher than 0.70, which implies a close relationship between these parameters (ABTS: ρ = 0.96; DPPH: ρ = 0.92; FRAP: ρ = 0.90; ORAC: ρ = 0.96; and Fe^2+^ chelating power: ρ = 0.70).

Numerous studies have reported promising antioxidant properties in hydrolyzates obtained from lungs of several livestock species, including bovine [57,58], chicken [59], and porcine [43,60,61,62], similar to those reported in this study. On the other hand, US pretreatment facilitates protein unfolding, which exposes new cleavage sites for enzymatic hydrolysis [7]. This fact can lead to a more intensive hydrolysis and a greater release of bioactive antioxidant peptides. The positive effect of the synergy of US pretreatment and Flavourzyme on improving antioxidant activities were previously reported on porcine liver [48], suggesting that this combination of techniques could be an interesting strategy to revalorize porcine organs and improve enzyme action efficiency.

Based on these findings, two specific conditions were selected for further analysis: (1) 1:100 E/S ratio hydrolyzates were the ones which showed most significant differences in antioxidant capacity, and (2) 1:20 E/S ratio hydrolyzates reported the best results in the release of taste-related free amino acids. These two conditions were subsequently chosen for peptidomic and in silico analysis of peptides identified by tandem mass spectrometry (MS/MS), with the aim of identifying the peptide profiles responsible for bioactivity and taste-related properties.

### 3.4. Peptidomics and In Silico Analysis

A total of 10,968 peptides from 4646 proteins were identified through peptidomic analysis. As shown in Table 4, samples with ultrasound pretreatment resulted in an increase in the number of total unique peptide sequences, suggesting that protein unfolding could improve enzyme accessibility to cleavage sites of the protein [7].

In PeptideRanker, values above 0.5 indicate a probability of being bioactive. In terms of bioactivity, when ultrasound pretreatment is performed, the number of peptide sequences identified with a score > 0.5 in PeptideRanker tool increased at 1:100 and 1:20 E/S ratio, whereas those with a score > 0.9 only increase at 1:20 E/S ratio. Regarding the results obtained using in silico taste-related bioinformatics tools (UMPred-FRL and BERT4Bitter), a higher amount of umami peptides compared to bitter was observed in all hydrolyzates (Table 4). These results suggest that these hydrolyzates can present a potential pleasant taste [46]. A cut-off of values above to 0.9 in PeptideRanker was chosen since they would have the highest probability of being bioactive. A total of 25 unique peptides with PeptideRanker scores > 0.9 were identified (Table 5) which means that they could potentially exert biological functions.

Some of the main biological functions that can exert these peptides are antioxidant and ACE, DPPIV, DPPIII, and TPPII inhibitory potential. ACE inhibitors control hypertension by reducing angiotensin II formation, leading to vasodilation. DPP-IV inhibitors can improve glucose regulation being helpful for type 2 diabetes, antioxidant could prevent cells damage by reducing the production of reactive oxygen species, DPP III inhibitors could exert opioid activity [63], and TPP II inhibitors may contribute to antitumor activity [64]. Regarding bioactivities, these peptides showed potential antioxidant activity scores (FRS Score) ranging from 0.43 to 0.64 according to the results obtained with AnOxPePred, which are in line with other studies on porcine slaughter by-products [65,66]. Additionally, 8 of 25 of the peptides present potential ACE inhibitory potential according to Deepstack-ACE tool, and 12 of 25 showed potential DPP-IV inhibition capacity according to StackDPPIV tool, which means that they could have potential to exert antihypertensive and antidiabetic activities. Toxicity and allergenicity analyses revealed potentially safety profiles: only 8 of 25 were potentially allergenic according to AllerTOP tool, and none were predicted as toxic according to ToxinPred. Furthermore, results for MLCPP tool showed that 14 of 25 peptide sequences were classified as cell-penetrating peptides (CPPs), highlighting their potential to exert their activities intracellularly. Taste-related peptides analysis by UMPred-FRL and BERT4Bitter indicated that 19 of 25 of these peptides present bitterness, and only 2 were umami. Taste attributes and bioactivity profiles can be closely related to their amino acid composition and physicochemical properties. CPPs generally show positive net charge, high amphipathicity, and low hydrophobicity [67]. These trends were confirmed in this study (Table S1) since most of CPPs present positive charge, neutral or positive amphipathicity, and low hydrophobicity with values lower than 0.1. In general, antioxidant peptides are characterized by small size and by the presence of hydrophobic (Leu (L), Val (V), Ala (A), Pro (P), Phe (F)) and aromatic (Tyr (Y), Trp (W)) amino acids, often located at the N-terminal. Meanwhile, acidic residues (Asp (D), Glu (E)) may contribute to metal-chelating activity [56]. For DPP-IV inhibition, the presence of P and A at the second N-terminal position is relevant [68], and short-chain amino acids (A, Cys (C), Gly (G), Ser (S), P, Asn (N), Thr (T), Val (V)) have been linked to ACE inhibition [63]. All these conditions can also be observed in Table S1 in the peptides that present these bioactivities.

In terms of taste, umami peptides are usually di- or tripeptides with acidic amino acids residues (D, E). In longer peptides (>8 amino acids), N- and C-terminal positioning of D/E, along with the presence of basic residues (H, K, R), can promote interaction with umami receptors T1R1/T1R3 [69]. In contrast, bitter peptides with more than 3 amino acids, usually bind with T2R receptor if their bulkiness is high and if their amino acid composition contains hydrophobic amino acids such as L, P or W [70]. These tendencies were observed in the peptides of this study, since bitter peptides are composed by hydrophobic amino acids, and both umami peptides identified (GDGWWGPGS and GDGWWGPGSRP) contained R and D residues (Table 5).

Hemoglobin, heat shock protein beta-1, and desmin are the main proteins of origin of these peptide sequences identified as potential bioactive by the PeptideRanker tool. These results align with those obtained by Bechaux et al. (2020) in a previous study, where hemoglobin was also the predominant protein in porcine lung [62]. These peptides should remain stable after gastrointestinal digestion to exert their bioactive function [71]. Thus, peptides with PeptideRanker > 0.9 were subjected to in silico gastrointestinal digestion simulation using BIOPEP-UWM, choosing pepsin, trypsin, and chymotrypsin as gastrointestinal proteases (Table 6).

A total of 35 fragments were released after this process, with 9 previously reported as bioactive in BIOPEP-UWM (Table 7). Antioxidant scores (0.53–0.37) according to AnOxPePred remained mostly stable in comparison with the undigested ones, while 2 of 9 showed ACE-inhibitory potential according to Deepstack-ACE, and all showed DPP-IV inhibitory potential according to StackDPPIV. These results suggest that the gastrointestinal fragments produced during in silico digestion would enhance the bioactivity potential of the peptides present in the native hydrolyzates, especially the antidiabetic activity. This pattern is in partial agreement with previous BIOPEP-UWM reports (Figure S1), which showed that 55.6% of these fragments have DPP-IV inhibitor potential, 55.5% ACE inhibitor potential, and 22.2% antioxidant, TPP II, and DPP III inhibitor potential. These findings are comparable to findings by Bechaux et al. (2020) (55% DPPIV, 31% ACE), who evaluated porcine lung hydrolyzates using papain and subtilisin. In fact, two of the fragments of this study (VY and PW) were also reported in this article, promoting the same bioactivities [62].

The obtained fragments after in silico gastrointestinal digestion are non-toxic, and 7 to 9 were predicted to be CPPs, which is crucial for exerting their biological function in the target tissue. These increase in CPPs are probably due to the function of the peptide transporter PEP-1 which facilitates the uptake of di- and tripeptides [72]. In this sense, peptides GIL and VY lacked cell-permeating properties probably due to the highest hydrophobicity that reduces their permeability to cell membrane. These findings suggest that gastrointestinal digestion could increase the bioavailability of peptides, since the fragments produced during in silico digestion have an enhanced cell-permeating capacity.

According to taste properties, the fragments retained a predominantly bitter profile (7 of 9) with only 1 being umami (DF), which may negatively affect overall taste. However, this analysis is qualitative, and actual impact depends on peptide thresholds and total content. Finally, Table S2 showed the 26 fragments not previously reported in BIOPEP database, with a 22 of 26 of the sequences with PeptideRanker scores > 0.5 and CPP potential according to MLCPP; 23 of 26 showed potential DPP-IV inhibitory activity according to StackDPPIV, and AnOxPePred antioxidant scores ranging from 0.58 to 0.38. All in all, these gastrointestinal fragments present several potential bioactivities that could probably be exerted due to their high cell-permeating capacity, suggesting that this hydrolyzates could exert a multifunctional bioactive effect helpful for the developing of functional ingredients.

## 4. Conclusions

In conclusion, US pretreatment in combination with Flavourzyme hydrolysis, especially at 1:20 ratio, seemed to be an interesting strategy to revalorize porcine lungs, developing hydrolyzates rich in pleasant free amino acids and umami peptides, with high antioxidant activity and with potential to exert multifunctional biological activities, such as ACE or DPPIV inhibition activities. The obtained results in this study could facilitate the development of an ingredient able to enhance pleasant tastes without adding salt or sugar and exert antioxidant activity in the incorporated product, making it more attractive and safer.

## Figures and Tables

**Table 1:** Physicochemical properties and nucleotides content of cooked porcine lung (*n* = 3; Mean ± SEM).

Properties and Composition	Weight (g)	pH	Water Activity (a_w_)	Moisture (g/100 g)	Protein (g/100 g)
	333.67 ± 13.43	7.03 ± 0.12	0.997 ± 0.002	78.04 ± 0.72	17.50 ± 0.28
Colour Properties	Luminosity (L*)	Redness (a*)	Yellowness (b*)	Chroma (C*)	Hue (°)
	32.57 ± 1.74	9.71 ± 0.23	6.91 ± 1.06	12.29 ± 0.85	35.50 ± 2.90
Nucleotides	Hypoxhantine	Inosine	AMP ^a^	IMP	GMP
Concentration (mg/100 g wet matter)	40.71 ± 0.95	5.79 ± 0.18	1.47 ± 0.02	1.71 ± 0.03	1.46 ± 0.02
Potential taste ^b^	Bitter ^(1)^	Bitter ^(5)^	Umami ^(1,2,3,4)^	Umami ^(1,2,3,4)^	Umami ^(1,2,3,4)^
Taste Threshold (mg/100 g wet matter) ^c^	-	-	50 ^(6,7,8)^	25 ^(6,7,8)^	12.5 ^(6,7,8)^
Taste Activity Value (TAV) (Dimensionless)	-	-	0.029 ± 0.000	0.069 ± 0.001	0.117 ± 0.002

^a^ AMP, IMP, and GMP refer to adenosine monophosphate, inosine monophosphate, and guanosine monophosphate, respectively. ^b^ Numbers between parentheses are the reference on taste attributes: ^(1)^ [13]; ^(2)^ [15]; ^(3)^ [19]; ^(4)^ [16]; and ^(5)^ [20]. ^c^ Numbers between parentheses are the reference on taste thresholds: ^(6)^ [23]; ^(7)^ [22]; and ^(8)^ [14].

**Table 2:** Degree of hydrolysis (%), free amino acid content (mg aas/ g wet matter), and Equivalent Umami Content (mg monosodium glutamate/100 g wet matter) of porcine lung hydrolyzates (*n* = 3; Mean ± SEM).

	Without US ^a^				With US			
**Enzyme:substrate ratio**	0	1:100	1:40	1:20	0	1:100	1:40	1:20
**Degree of hydrolysis (%)**	2.42 ± 0.18 fDX	15.90 ± 2.40 eCY	36.04 ± 1.28 cdBY	43.78 ± 2.46 abAY	3.67 ± 1.24 fGX	29.72 ± 2.53 dFX	42.55 ± 1.18 bcEX	49.96 ± 1.37 aEX
**FAA content (mg aas/g wet matter)**								
Asp (Umami) ^(1,2,3,4) b^	0.51 ± 0.06 eCY ^e^	1.09 ± 0.03 cdeBCY	1.54 ± 0.12 cdBY	3.57 ± 0.26 bAY	0.73 ± 0.06 deGX	1.43 ± 0.03 cdeFGX	1.89 ± 0.08 cFX	4.72 ± 0.47 aEX
Glu (Umami) ^(1,2,3,4)^	1.72 ± 0.09 dBY	3.14 ± 0.18 cdBY	3.34 ± 0.12 cdBX	6.88 ± 0.77 bAY	2.58 ± 0.21 cdFX	3.59 ± 0.08 cdFX	4.08 ± 0.43 cFX	9.16 ± 0.86 aEX
**Umami aas**	**2.23 ± 0.14 dCY**	**4.23 ± 0.15 cdBCY**	**4.89 ± 0.24 cdBY**	**10.45 ± 1.81 bAY**	**3.31 ± 0.30 cdFX**	**5.02 ± 0.11 cdFX**	**5.98 ± 0.59 cFX**	**13.87 ± 2.30 aEX**
Ser (Sweet) ^(1,2,3,4),^	0.37 ± 0.07 eCX	1.41 ± 0.10 cdeCY	2.49 ± 0.25 bcBY	5.94 ± 0.42 aAX	0.51 ± 0.06 deGX	1.94 ± 0.08 bcdFGX	3.09 ± 0.03 bFX	7.17 ± 0.72 aEX
Gly (Sweet) ^(1,2,3,4)^	1.13 ± 0.12 cCY	1.84 ± 0.02 bcBC	2.26 ± 0.15 bcBY	4.70 ± 0.39 aAY	1.68 ± 0.15 bcFX	2.21 ± 0.08 bcFX	2.75 ± 0.08 bFX	5.93 ± 0.58 aEX
Gln (Sweet) ^(4)^	0.27 ± 0.02 cCY	1.85 ± 0.21 bcBCY	2.78 ± 0.14 bBY	6.70 ± 0.57 aAY	0.42 ± 0.03 cGX	2.30 ± 0.20 bFX	3.43 ± 0.16 bFX	8.02 ± 0.81 aEX
Thr (Sweet) ^(1,2,3,4)^	0.18 ± 0.03 DdY	1.25 ± 0.10 cdCY	2.22 ± 0.18 bcBY	5.38 ± 0.39 aAY	0.36 ± 0.04 dGX	1.70 ± 0.07 bcFGX	2.74 ± 0.03 bFX	6.49 ± 0.65 aEX
Ala (Sweet) ^(1,2,3,4)^	0.48 ± 0.54 dCY	2.00 ± 0.22 cdeBY	3.29 ± 0.30 bcBY	7.84 ± 0.61 aAY	0.79 ± 0.07 deGX	2.68 ± 0.09 bcdFGX	4.09 ± 0.06 bFX	9.46 ± 0.97 aEX
**Sweet aas**	**2.43 ± 0.707 dCY**	**8.33 ± 0.65 bcdBY**	**13.03 ± 1.01 bBY**	**30.57 ± 4.20 aAY**	**3.75 ± 0.35 cdGX**	**10.84 ± 0.52 bcFX**	**16.09 ± 0.36 bFX**	**37.06 ± 6.46 aEX**
Arg (Bittersweet) ^(2,3)^	0.19 ± 0.04 dCX	2.37 ± 0.33 cdBY	4.09 ± 0.21 bcBY	9.44 ± 0.74 aAX	0.28 ± 0.06 dGX	3.25 ± 0.15 bcFGX	4.96 ± 0.19 bFX	11.33 ± 1.01 aEX
Pro (Bittersweet) ^(2,3)^	0.32 ± 0.09 bBX	0.38 ± 0.01 bBY	0.37 ± 0.10 bBX	1.10 ± 0.15 aAX	0.48 ± 0.05 bFX	0.44 ± 0.01 bFX	0.50 ± 0.03 bFX	1.31 ± 0.19 aEX
Val (Bittersweet) ^(2,3)^	0.25 ± 0.05 eCX	1.82 ± 0.18 cdeBCY	3.17 ± 0.29 bcBY	8.11 ± 0.68 aAX	0.36 ± 0.04 deGX	2.50 ± 0.08 bcdFGX	4.00 ± 0.05 bFX	9.81 ± 1.01 aEX
Lys (Bittersweet) ^(2,3)^	0.62 ± 0.15 dCY	2.59 ± 0.26 cdBCY	4.78 ± 0.39 bcBY	11.13 ± 0.78 aAY	0.85 ± 0.12 dGX	3.65 ± 0.18 bcFGX	5.86 ± 0.07 bFX	13.35 ± 1.17 aEX
Met (Bittersweet) ^(2,3)^	0.08 ± 0.03 dBX	0.65 ± 0.04 bcdBY	1.28 ± 0.16 bcBY	3.57 ± 0.52 aAX	0.14 ± 0.01 cdGX	0.96 ± 0.05 bcdFGX	1.65 ± 0.05 bFX	4.25 ± 0.43 aEX
**Bittersweet aas**	**1.47 ±0.59 dCX**	**7.81 ± 0.79 cdBY**	**13.69 ± 1.15 bcBY**	**33.35 ± 5.03 aAX**	**2.10 ± 0.17 dGX**	**10.79 ± 0.45 bcFX**	**16.79 ± 0.46 bFX**	**40.04 ± 6.61 aEX**
Tau (Bitter) ^(1,2)^	1.52 ± 0.14 cBY	1.79 ± 0.017 cBX	1.70 ± 0.14 cBX	2.80 ± 0.25 abAY	2.14 ± 0.24 bcFX	2.04 ± 0.06 bcFX	1.98 ± 0.05 cFX	3.47 ± 0.28 aEX
His (Bitter) ^(1,3,4)^	0.06 ± 0.02 dCX	0.63 ± 0.07 cdBCY	1.37 ± 0.17 bcBY	3.79 ± 0.34 aAX	0.07 ± 0.01 dGX	0.93 ± 0.03 bcdFGX	1.77 ± 0.02 bFX	4.57 ± 0.51 aEX
Tyr (Bitter) ^(1,2,3,4)^	0.12 ± 0.02 eCX	1.00 ± 0.07 cdeBCY	1.81 ± 0.18 bcBY	4.67 ± 0.40 aAX	0.09 ± 0.04 eGX	1.39 ± 0.19 bcdFGX	2.30 ± 0.06 bFX	5.61 ± 0.59 aEX
Ile (Bitter) ^(1,2,3,4)^	0.12 ± 0.02 dCY	1.03 ± 0.06 cdBY	1.77 ± 0.11 bcBY	4.28 ± 0.35 aAY	0.20 ± 0.03 dGX	1.41 ± 0.06 bcFX	2.19 ± 0.06 bFX	5.35 ± 0.54 aEX
Leu (Bitter) ^(1,2,3,4)^	0.33 ± 0.07 dCY	3.94 ± 0.25 cdCX	6.62 ± 0.61 bcBY	15.63 ± 1.29 aAY	0.53 ± 0.05 dHX	5.23 ± 0.18 bcGX	8.27 ± 0.16 bFX	18.25 ± 1.77 aEX
Phe (Bitter) ^(1,2,3,4)^	0.14 ± 0.03 dCY	1.81 ± 0.14 cdCY	3.61 ± 0.30 bcBY	7.36 ± 0.63 aAY	0.21 ± 0.02 dFX	2.38 ± 0.15 cFX	4.43 ± 0.10 bFX	9.13 ± 0.84 aEX
Trp (Bitter) ^(1,2,3,4)^	0.06 ± 0.01 dCY	0.50 ± 0.05 cdBY	0.98 ± 0.12 bcBY	2.24 ± 0.18 aAY	0.08 ± 0.00 dGX	0.65 ± 0.05 cFGX	1.22 ±0.02 bFX	2.63 ± 0.23 aEX
**Bitter aas**	**2.36 ± 0.39 dCY**	**10.71 ± 0.48 cdBCY**	**17.87 ± 1.63 bcBY**	**40.78 ± 6.03 aAY**	**3.32 ± 0.31 dGX**	**14.02 ± 0.73 bcFGX**	**22.15 ± 0.41 bFX**	**49.01 ± 8.25 aEX**
Hyp	0.02 ± 0.00 cBY	0.01 ± 0.00 cBX	0.03 ± 0.01 bcBX	0.09 ± 0.03 abAY	0.03 ± 0.01 bcFX	0.03 ± 0.01 bcFX	0.03 ± 0.01 bcFX	0.13 ± 0.03 aEX
Asn	0.10 ± 0.02 eDY	0.99 ± 0.09 cdeCY	1.91 ± 0.11 bcBY	4.77 ± 0.35 aDY	0.16 ± 0.01 deGX	1.41 ± 0.05 bcdFGX	2.37 ± 0.07 bFX	5.96 ± 0.64 aEX
Orn	0.14 ± 0.03 cCX	0.18 ± 0.00 bcBCY	0.21 ± 0.04 bcBY	0.33 ± 0.01 aAX	0.20 ± 0.02 bcGX	0.22 ± 0.01 bFX	0.23 ± 0.02 bFX	0.36 ± 0.02 aEX
**Taste aas**	**8.41 ± 1.61 eCY**	**31.07 ± 1.77 cdeBCY**	**49.48 ± 4.03 bcBY**	**115.14 ± 17.06 aAY**	**12.48 ± 1.13 deGX**	**40.67 ± 1.81 bcdFGX**	**61.18 ± 1.59 bFX**	**139.99 ± 23.61 aEX**
**EAA ^c^**	**1.85 ± 0.60 dCX**	**14.22 ± 1.14 cdCY**	**25.78 ± 2.32 bcBY**	**61.49 ± 9.04 aAX**	**2.80 ± 0.32 dGX**	**19.40 ± 0.85 bcFX**	**32.12 ± 0.46 bFX**	**73.83 ± 12.38 aEX**
**BCAA ^c^**	**0.71 ± 0.20 dCY**	**6.79 ± 0.49 cdBY**	**11.56 ± 1.01 bcBY**	**28.02 ± 4.08 aAY**	**1.09 ± 0.11 dGX**	**9.13 ± 0.33 bcFX**	**14.46 ± 0.27 bFX**	**33.41 ± 5.76 aEX**
**Total aas**	**10.51 ± 1.94 dCY**	**34.33 ± 1.52 bcdBCY**	**53.60 ± 4.37 bBY**	**124.03 ± 18.64 aAY**	**15.37 ± 1.51 cdGX**	**44.70 ± 1.91 bcFGX**	**66.07 ± 1.62 bFX**	**151.20 ± 25.55 aEX**
% taste ^d^	80.66 ± 0.56 cCX	89.87 ± 1.78 bBX	92.31 ± 0.11 aAX	92.85 ± 0.19 aAX	81.27 ± 0.72 cGX	90.98 ± 0.16 abFX	92.59 ± 0.17 aEX	92.59 ± 0.04 aEX
% EAA ^d^	14.99 ± 2.27 cCX	32.68 ± 4.76 bBX	42.18 ± 0.36 aBX	43.05 ± 0.14 aAX	15.83 ± 0.24 cGX	37.80 ± 0.11 abFX	42.57 ± 0.48 aEX	42.35 ± 0.13 aEX
%BCAA ^d^	6.64 ± 0.68 eCX	20.77 ± 0.48 cdBX	21.56 ± 0.13 bcABX	22.61 ± 0.11 aAX	7.11 ± 0.06 eGX	20.43 ± 0.14 dFX	21.88 ± 0.14 abEX	22.09 ± 0.08 aEX
**EUC (g MSG/100 g wet matter)**								
	**11.61 ± 0.73 dCY**	**21.25 ± 0.95 cdBY**	**22.86 ± 1.14 cdBX**	**47.19 ± 8.93 bAY**	**17.37 ± 1.33 cdGX**	**24.40 ± 0.85 cdFX**	**27.94 ± 3.39 cFX**	**62.85 ± 10.41 aEX**

^a^ US, MSG, FAA, and EUC refer to ultrasound pretreatment, monosodium glutamate, free amino acids, and equivalent umami concentration, respectively. ^b^ The numbers indicate the reference taken into account to establish the taste attribute: ^(1)^ [13], ^(2)^ [15], ^(3)^ [14], ^(4)^ [16]. ^c^ The summatory of Threonine (Thr), Lysine (Lys), Isoleucine (Ile), Leucine (Leu), Valine (Val), Tryptophan (Trp), Histidine (His), and Methionine (Met) was expressed as the total essential amino acids content (EAAs), while the summatory of Ile, Leu, and Val correspond to the branched-chain amino acid content (BCAAs). ^d^ The percentage was obtained by dividing the total content of taste related essential and branched-chain amino acids by the total content of amino acids. ^e^ Different lowercase letters (a, b, c, d, e, f) denote significant differences among all samples. Different uppercase letters (A, B, C, D) denote significant differences within the group of samples without ultrasound pretreatment. Different uppercase letters (E, F, G, H) denote significant differences within the group of samples with ultrasound pretreatment. Different uppercase letters (X, Y) indicate significant differences between pairs of samples with the same enzyme: substrate ratio (0, 1/100, 1/40, 1/20).

**Table 3:** Taste active values (dimensionless) of free amino acids of porcine lung hydrolyzates. TAV > 1 = ◼; TAV > 5 = ◼; TAV > 10 = ◼; TAV > 15 = ◼; TAV > 20 = ◼.

Taste Activity Values		Without US ^a^				With US			
Enzyme:Substrate Ratio (E/S)		0	1:100	1:40	1:20	0	1:100	1:40	1:20
Amino Acid	Taste Threshold (mg/g)								
Asp	1.0 ^b (1,2)^	0.51	1.09	1.54	3.57	0.73	1.43	1.89	4.72
Glu	0.3 ^(1,2,3)^	5.73	10.46	11.15	22.93	8.59	11.95	13.61	30.52
Ser	1.5 ^(1,2,3)^	0.25	0.94	1.66	3.96	0.34	1.29	2.06	4.78
Gly	1.3 ^(1,2,3)^	0.87	1.41	1.74	3.62	1.29	1.70	2.11	4.56
Thr	2.6 ^(1,2,3)^	0.07	0.48	0.85	2.07	0.14	0.66	1.05	2.50
Ala	0.6 ^(1,2,3)^	0.80	3.33	5.49	13.07	1.32	4.47	6.82	15.76
Arg	0.5 ^(1,2,3)^	0.37	4.74	8.19	18.88	0.55	6.50	9.92	22.65
Pro	3.0 ^(1,2,3)^	0.11	0.13	0.12	0.37	0.16	0.15	0.17	0.44
Val	0.4 ^(1,2,3)^	0.62	4.56	7.91	20.28	0.90	6.24	9.99	24.52
Met	0.3 ^(1,2,3)^	0.28	2.16	4.25	11.89	0.46	3.19	5.50	14.15
Lys	0.5 ^(1,2,3)^	1.25	5.17	9.55	22.26	1.69	7.29	11.72	26.71
Tau	18.8 ^(3)^	0.08	0.10	0.09	0.15	0.11	0.11	0.11	0.19
His	0.2 ^(1,2,3)^	0.32	3.17	6.84	18.96	0.36	4.63	8.84	22.85
Tyr	0.9 ^(3)^	0.12	1.04	1.88	4.84	0.10	1.44	2.38	5.80
Ile	0.9 ^(1,2,3)^	0.14	1.14	1.97	4.76	0.22	1.56	2.44	5.94
Leu	1.9 ^(1,2,3)^	0.18	2.08	3.48	8.22	0.28	2.75	4.35	9.61
Phe	0.9 ^(1,2,3)^	0.15	2.02	4.01	8.18	0.23	2.64	4.92	10.15
Trp	0.9 ^(3)^	0.07	0.56	1.09	2.48	0.09	0.72	1.35	2.92

^a^ US and TAV refer to ultrasound pretreatment and taste activity value, respectively. ^b^ The numbers indicate the reference taken into account to establish the taste attribute: ^(1)^ [14], ^(2)^ [15], ^(3)^ [13].

**Table 4:** Total number of unique peptide sequences identified by MS/MS in the studied samples and in silico evaluation of bioactive potential and taste of porcine lung hydrolyzates.

Enzyme:Substrate Ratio	1:100	1:100 Ultrasound	1:20	1:20 Ultrasound
Total Peptides	2792	2856	2520	2800
Bioactive peptides > 0.5 ^a^	277	305	317	392
Bioactive peptides > 0.9	10	10	13	18
Umami peptides ^b^	1511	1502	1188	1299
Bitter peptides ^b^	851	843	762	790

^a^ Peptide bioactivity was ranked using the in silico tool PeptideRanker (http://distilldeep.ucd.ie/PeptideRanker, accessed on 19 July 2025). Scores above 0.5 indicate potential bioactivity. ^b^ Umami peptides were evaluated using the in silico tool UMPred-FRL (https://pmlabstack.pythonanywhere.com/UMPred-FRL, accessed on 19 July 2025) and the bitter peptides using BERT4Bitter (https://pmlab.pythonanywhere.com/BERT4Bitter, accessed on 19 July 2025).

**Table 5:** In silico evaluation of potential biological properties and taste of bioactive peptides ranked above 0.9 on PeptideRanker.

Nº	Sequence	PRK ^a^	FRS Score	CHEL Score	Allergencity	Toxicity	CPP	AMP	ACE	DPPIV	Umaminess	Bitterness
1	GFDPFLF	0.991	0.46	0.25	Yes	No	No	No	No	Yes	No	Yes
2	PPPFFPPRLPP	0.971	0.59	0.30	No	No	Yes	No	No	No	No	Yes
3	ADHPFLF	0.970	0.51	0.29	No	No	No	No	No	No	No	No
4	GWPLPPPYP	0.963	0.64	0.30	No	No	Yes	No	Yes	No	No	No
5	YPWTQRFF	0.956	0.48	0.20	Yes	No	Yes	No	Yes	No	No	Yes
6	RPPPFFPPRLPP	0.953	0.60	0.29	No	No	Yes	No	No	Yes	No	Yes
7	GGGGGGGGGGGLGGGLG	0.951	0.52	0.17	No	No	No	No	No	Yes	No	Yes
8	GDSWGILF	0.951	0.47	0.22	No	No	No	No	No	No	No	No
9	WDPFRDWYP	0.948	0.48	0.21	Yes	No	Yes	No	Yes	Yes	No	Yes
10	GAPSFPLG	0.942	0.44	0.25	No	No	No	No	No	Yes	No	Yes
11	GDGWWGPGSRP	0.939	0.63	0.23	No	No	Yes	No	Yes	No	Yes	Yes
12	GGGGGGGGGGGLGGGLGN	0.937	0.49	0.16	No	No	No	No	No	Yes	No	Yes
13	GDGWWGPGS	0.930	0.62	0.20	No	No	Yes	No	Yes	No	Yes	Yes
14	WDPFRDWYPA	0.928	0.53	0.20	Yes	No	Yes	No	Yes	No	No	No
15	FGGAPSFPL	0.926	0.48	0.24	No	No	No	No	No	Yes	No	Yes
16	FPDPPPLSPPVLG	0.925	0.46	0.30	No	No	Yes	No	No	No	No	No
17	DFLGDSWGILF	0.925	0.43	0.22	Yes	No	No	No	No	No	No	Yes
18	GGAPSFPLGSPL	0.925	0.46	0.26	No	No	Yes	No	No	Yes	No	Yes
19	GPPDPILG	0.922	0.43	0.25	No	No	No	No	No	Yes	No	Yes
20	VYPWTQRFF	0.913	0.50	0.20	Yes	No	Yes	No	Yes	No	No	Yes
21	GPSGPPGLP	0.912	0.57	0.28	No	No	Yes	No	No	Yes	No	No
22	SPSWDPFRDWYPAH	0.911	0.47	0.22	Yes	No	Yes	No	Yes	No	No	No
23	GPPDPIL	0.907	0.46	0.27	No	No	No	No	No	Yes	No	Yes
24	GNNTPIFF	0.906	0.44	0.24	Yes	No	Yes	No	No	No	No	Yes
25	GAGGPGAGGFG	0.900	0.50	0.23	No	No	No	No	No	Yes	No	Yes

^a^ Potential biological properties and taste were analyzed using the following tools: bioactivity rank from PeptideRanker (http://distilldeep.ucd.ie/PeptideRanker/, accessed on 19 July 2025), free radical scavening (FRS) and chelating (CHEL) scores from AnOxPePred—1.0 (https://services.healthtech.dtu.dk/services/AnOxPePred-1.0/ accessed on 19 July 2025), allergenicity from AllerTOP v.2.0(https://ddg-pharmfac.net/allertop_test/, accessed on 19 July 2025), toxicity from ToxinPred (https://webs.iiitd.edu.in/raghava/toxinpred/, accessed on 19 July 2025), cell-permeating potential (CPP) from MLCPP (http://www.thegleelab.org/MLCPP/MLCPP.html, accessed on 19 July 2025), antimicrobial power (AMP) from DBAASP (https://dbaasp.org/tools?page=linear-amp-prediction, accessed on 19 July 2025), Angiotensin Converting Enzyme (ACE) Inhibition potential from Deepstack-ACE (https://pmlabqsar.pythonanywhere.com/predict_DeepstackACE, accessed on 19 July 2025), and Dipeptidyl peptidase IV inhibition potential from StackDPPIV (https://pmlabstack.pythonanywhere.com/StackDPPIV, accessed on 19 July 2025). For taste prediction, umami taste was evaluated with the UMPred-FRL tool (https://pmlabstack.pythonanywhere.com/UMPred-FRL, accessed on 19 July 2025) and bitter taste with BERT4Bitter (https://pmlab.pythonanywhere.com/BERT4Bitter, accessed on 19 July 2025).

**Table 6:** Protein of origin and results of in silico gastrointestinal digestion of bioactive peptides ranked above 0.9 on PeptideRanker.

Nº	Sequence	Protein of Origin	Hydrolyzate	In Silico Gastrointestinal Digestion ^a^
1	GFDPFLF	Xaa-Pro aminopeptidase 2	1:20 US	**GF ^b^**—DPF—L—F -
2	PPPFFPPRLPP	Antibacterial protein PR-39	1:100, 1:100 US, 1:20	**PPPF**—F—PPR—L—PP
3	ADHPFLF	Leukocyte elastase inhibitor	1:20 US	ADH—PF—L—F -
4	GWPLPPPYP	Tryptase	1:20 US	**GW**—**PL**—PPPY—P
5	YPWTQRFF	Hemoglobin subunit beta	1:100, 1:100 US, 1:20 US	Y—**PW**—TQR—F—F -
6	RPPPFFPPRLPP	Antibacterial protein PR-39	1:20, 1:20 US	R—**PPPF**—F—PPR—L—PP
7	GGGGGGGGGGGLGGGLG	Calpain small subunit 1	1:100, 1:100 US, 1:20, 1:20 US	GGGGGGGGGGGL—GGGL—G
8	GDSWGILF	Peroxiredoxin-6	1:100, 1:100 US, 1:20	GDSW—**GIL**—F -
9	WDPFRDWYP	Heat shock protein beta-1	1:100 US	W—DPF—R—DW—Y—P
10	GAPSFPLG	Desmin	1:100	GAPSF—**PL**—G
11	GDGWWGPGSRP	Epoxide hydrolase 1	1:20 US	GDGW—W—GPGSR—P
12	GGGGGGGGGGGLGGGLGN	Calpain small subunit 1	1:100, 1:100 US, 1:20, 1:20 US	GGGGGGGGGGGL—GGGL—GN -
13	GDGWWGPGS	Epoxide hydrolase 1	1:20	GDGW—W—GPGS
14	WDPFRDWYPA	Heat shock protein beta-1	1:20, 1:20 US	W—DPF—R—DW—Y—PA
15	FGGAPSFPL	Desmin	1:20	F—GGAPSF—**PL** -
16	FPDPPPLSPPVLG	ATP-binding cassette sub-family F member 1	1:20 US	F—PDPPPL—SPPVL—G
17	DFLGDSWGILF	Peroxiredoxin-6	1:100	**DF**—L—GDSW—**GIL**—F -
18	GGAPSFPLGSPL	Desmin	1:100, 1:100 US, 1:20, 1:20 US	GGAPSF—**PL**—GSPL -
19	GPPDPILG	Aspartate aminotransferase, mitochondrial	1:100, 1:100 US, 1:20, 1:20 US	GPPDPIL—G
20	VYPWTQRFF	Hemoglobin subunit beta	1:20 US	**VY**—**PW**—TQR—F—F -
21	GPSGPPGLP	Complement C1q subcomponent subunit A	1:20 US	GPSGPPGL—P
22	SPSWDPFRDWYPAH	Heat shock protein beta-1	1:100 US, 1:20 US	SPSW—DPF—R—DW—Y—PAH -
23	GPPDPIL	Aspartate aminotransferase, mitochondrial	1:20, 1:20 US	GPPDPIL-
24	GNNTPIFF	Catalase	1:20, 1:20 US	GN—N—TPIF—F -
25	GAGGPGAGGFG	Heat shock 70 kDa protein 1B	1:100, 1:100 US, 1:20, 1:20 US	GAGGPGAGGF—G

^a^ Results of the combined enzyme action of pepsin (pH 1.3, EC 3.4 23 1), trypsin (EC 3.4 21 4), and chymotrypsin C (EC 3.4 21 2) in silico gastrointestinal digestion performed in BIOPEP-UWM (https://biochemia.uwm.edu.pl/biopep/start_biopep.php, accessed on 19 July 2025). ^b^ Peptide fragments in bold refer to peptides whose biological activity is currently reported and registered in BIOPEP-UWM database.

**Table 7:** In silico analysis of biological, physicochemical, and taste properties of fragments obtained during in silico gastrointestinal digestion with biological activity reported in BIOPEP-UWM database.

Nº	1	2	3	4	5	6	7	8	9
Digestion fragment	PPPF	PW	GW	DF	GF	PF	GIL	PL	VY
Location	P2, P6	P5, P20	P4	P17	P1	P3	P8, P17	P4, P10, P15, P18	P20
Peptide Ranker score ^a^	0.96	0.95	0.95	0.94	0.93	0.92	0.61	0.51	0.1
Toxicity	No	No	No	No	No	No	No	No	No
CPP	Yes	Yes	Yes	Yes	Yes	Yes	No	Yes	No
FRS score	0.5	0.53	0.52	0.38	0.44	0.45	0.37	0.43	0.49
CHEL score	0.32	0.28	0.26	0.28	0.28	0.3	0.25	0.31	0.24
ACE	No	Yes	No	No	No	No	No	No	Yes
DPPIV	Yes	Yes	Yes	Yes	Yes	Yes	Yes	Yes	Yes
Umaminess	No	No	No	Yes	No	No	No	No	No
Bitterness	Yes	Yes	Yes	Yes	Yes	Yes	No	No	Yes
Taste BIOPEP	x	x	x	x	Bitter	Bitter	x	Bitter	Bitter
SVM Score	−0.84	−0.63	−0.7	−0.8	−0.87	−0.87	−0.79	−0.7	−0.8
Hydrophobicity	0.07	0.07	0.13	−0.05	0.19	0.14	0.47	0.12	0.28
Steric hindrance	0.3	0.21	0.3	0.73	0.34	0.27	0.64	0.22	0.7
Sidebulk	0.3	0.21	0.3	0.73	0.34	0.27	0.64	0.22	0.7
Hydropathicity	−0.33	−0.62	−0.33	−0.35	0.6	0.3	2.63	0.55	1.45
Amphipathicity	0	0	0	0	0	0	0	0	0
Hydrophilicity	−0.42	−0.85	−0.85	0.25	−0.62	−0.62	−1.2	−0.45	−1.9
Net Hydrogen	0	0.25	0.25	0.5	0	0	0	0	0.5
Charge	0	0	0	−1	0	0	0	0	0
pI	5.88	5.88	5.88	3.8	5.88	5.88	5.88	5.88	5.88
Mol wt	750.96	673.82	633.76	280.29	516.64	556.7	301.43	454.67	280.34
Bioactivities Reported ^b^	DPPCP ^c^	ANTIOX	ACE	ACE	ACE	DPPIV	ACE	ACE	ACE
		DPPIV	DPPIV	GCII	DPPIV	DPPIII		DPPIV	ANTIOX
			ANTIOX		DPPIII	ACE2		XAA	DPPIV
			TPPII		TPPII			LCP	DPPIII
					ACYLP				TPPII

^a^ Potential biological properties and physicochemical properties were evaluated using the following tools: bioactivity rank from PeptideRanker (http://distilldeep.ucd.ie/PeptideRanker/, accessed on 19 July 2025), toxicity and potential physicochemical properties (SVM Score, hydrophobicity, steric hindrance, sidebulk, hydropathicity, amphipathicity, hydrophilicity, net hydrogen, charge, isoelectric point (pI), Molecular weight (Mol wt)) from ToxinPred (https://webs.iiitd.edu.in/raghava/toxinpred/, accessed on 19 July 2025), free radical scavening (FRS) and chelating (CHEL) scores from AnOxPePred—1.0 (https://services.healthtech.dtu.dk/services/AnOxPePred-1.0/, accessed on 19 July 2025), and cell-permeating power (CPP) from MLCPP (http://www.thegleelab.org/MLCPP/MLCPP.html, accessed on 19 July 2025). Dipeptidyl peptidase IV inhibition potential from StackDPPIV (https://pmlabstack.pythonanywhere.com/StackDPPIV, accessed on 19 July 2025) and Angiotensin Converting Enzyme (ACE) Inhibition potential from Deepstack-ACE (https://pmlabqsar.pythonanywhere.com/predict_DeepstackACE, accessed on 19 July 2025). Umami taste was evaluated using the tool UMPred-FRL (https://pmlabstack.pythonanywhere.com/UMPred-FRL, accessed on 19 July 2025), and bitter taste using the tool BERT4Bitter (https://pmlab.pythonanywhere.com/BERT4Bitter, accessed on 19 July 2025). Finally, taste attributes were extracted from BIOPEP-UWM database (https://biochemia.uwm.edu.pl/biopep/start_biopep.php, accessed on 19 July 2025). ^b^ The biological activities previously reported in BIOPEP were compiled using the following abbreviations. (https://biochemia.uwm.edu.pl/biopep/start_biopep.php, accessed on 19 July 2025). ^c^ The acronyms stand for the following terms: *DPPCP*: Dipeptidyl carboxypeptidase inhibitor; *Antiox*: Antioxidant; *DPPIV:* Dipeptidyl peptidase IV inhibitor; *ACE*: Angiotensin Converting Enzyme Inhibitor; *TPPII*: tripeptidyl peptidase II inhibitor; *GCII*: Glutamate carboxypeptidase II inhibitor; *DPPIII:* Dipeptidyl peptidase III inhibitor; *ACYLP*: Acylaminoacyl peptidase inhibitor; *ACE2*: Angiotensin Converting Enzyme 2 Inhibitor; *XAA*: Xaa-Pro aminopeptidase inhibitor; and *LCP:* Lactocepin inhibitor.

## Data Availability

The raw data supporting the conclusions of this article will be made available by the authors on request.

## Supplementary Materials

The following supporting information can be downloaded at https://www.mdpi.com/article/10.3390/foods14183243/s1, Table S1: In silico evaluation of potential physicochemical properties of bioactive peptides ranked above 0.9 on PeptideRanker. Figure S1: Potential biological activities expressed in percentages of fragments obtained during in silico gastrointestinal digestion with biological activity re-ported in BIOPEP-UWM database. Table S2: In silico evaluation of biological and taste properties of fragments obtained during in silico gastrointestinal digestion with nonbiological activity reported in BIOPEP-UWM database.
