# Supplementary material for: Impact of Ultrasound Pretreatment and Enzyme Concentration on Taste and Biological Activities of Porcine Lung Hydrolyzates
Source: Foods. 2025 Sep 18;14(18):3243. doi: 10.3390/foods14183243 (PMC12469255; doi:10.3390/foods14183243)
Supplement: Supplementary file 1 [file foods-14-03243-s001.zip › foods-3859369-supplementary.pdf]

Supplementary material

Table S1. In-silico evaluation of potential physicochemical properties of bioactive peptides ranked above 0.9 on PeptideRanker

Nº	Sequence	SVM Score[a]	Hydro phobicity	Steric hindrance	Sidebulk	Hydropathicity	Amphipathicity	Hydrophilicity	Net Hydrogen	Charge	pI	Mol wt
1	GFDPFLF	-0.82	0.25	0.63	0.63	0.96	0	-0.9	0.14	-1	3.8	842.05
2	PPPFFPPRLPP	-0.72	-0.05	0.47	0.47	-0.57	0.22	-0.35	0.36	1	10.11	1261.67
3	ADHPFLF	-0.37	0.12	0.51	0.51	0.41	0.21	-0.69	0.29	-0.5	5.09	846.04
4	GWPLPPPYP	-0.17	0.08	0.47	0.47	-0.76	0	-0.83	0.22	0	5.88	1023.32
5	YPWTQRFF	-0.58	-0.14	0.61	0.61	-0.86	0.46	-0.99	1.12	1	9.1	1144.4
6	RPPPFFPPRLPP	-0.77	-0.19	0.48	0.48	-0.9	0.41	-0.07	0.67	2	12.01	1417.87
7	GGGGGGGGGGLGGGLG	-0.84	0.2	0.66	0.66	0.09	0	-0.21	0	0	5.88	1100.41
8	GDSWGILF	-1.15	0.2	0.64	0.64	0.64	0	-0.78	0.38	-1	3.8	894.11
9	WDPFRDWYP	-0.5	-0.22	0.59	0.59	-1.67	0.27	-0.29	1	-1	4.21	1281.5
10	GAPSFPLG	-1.02	0.16	0.54	0.54	0.45	0	-0.56	0.12	0	5.88	744.95
11	GDGWWGPGSRP	-0.65	-0.14	0.58	0.58	-1.4	0.22	-0.05	0.73	0	6.19	1171.39
12	GGGGGGGGGGLGGGLGN	-0.84	0.16	0.67	0.67	-0.11	0	-0.19	0.11	0	5.88	1214.53
13	GDGWWGPGS	-0.87	0.04	0.6	0.6	-1.03	0	-0.39	0.44	-1	3.8	918.06
14	WDPFRDWYPA	-0.64	-0.17	0.58	0.58	-1.32	0.25	-0.31	0.9	-1	4.21	1352.59
15	FGGAPSFPL	-1.04	0.21	0.56	0.56	0.71	0	-0.78	0.11	0	5.88	892.14
16	FPDPPPLSPPVLG	-0.51	0.07	0.51	0.51	0.02	0	-0.33	0.15	-1	3.8	1332.74
17	DFLGDSWGILF	-1.41	0.18	0.64	0.64	0.75	0	-0.68	0.36	-2	3.57	1269.58
18	GGAPSFPLGSPL	-1.16	0.14	0.54	0.54	0.38	0	-0.5	0.17	0	5.88	1099.42
19	GPPDPILG	-0.29	0.08	0.55	0.55	-0.1	0	-0.08	0.12	-1	3.8	764.99
20	VYPWTQRFF	-0.76	-0.06	0.62	0.62	-0.3	0.41	-1.04	1	1	9.1	1243.55
21	GPSGPPGLP	-0.25	0.05	0.5	0.5	-0.51	0	-0.17	0.11	0	5.88	778
22	SPSWDPFRDWYPAH	-0.69	-0.19	0.52	0.52	-1.4	0.28	-0.21	0.86	-0.5	5.22	1761.06
23	GPPDPIL	-0.24	0.07	0.54	0.54	-0.06	0	-0.09	0.14	-1	3.8	707.92
24	GNNTPIFF	-1	0.07	0.65	0.65	0.05	0	-0.85	0.62	0	5.88	909.12
25	GAGGPGAGGFG	-0.9	0.2	0.62	0.62	0.18	0	-0.32	0	0	5.88	803.99

[a] Potential physicochemical properties (SVM Score, hydrophobicity, steric hindrance, sidebulk, hydropathicity, amphipathicity, hydrophilicity, net hydrogen, charge, isoelectric point (pI), Molecular weight (Mol wt) were extracted from ToxinPred (http://crdd.osdd.net/raghava/toxinpred/).

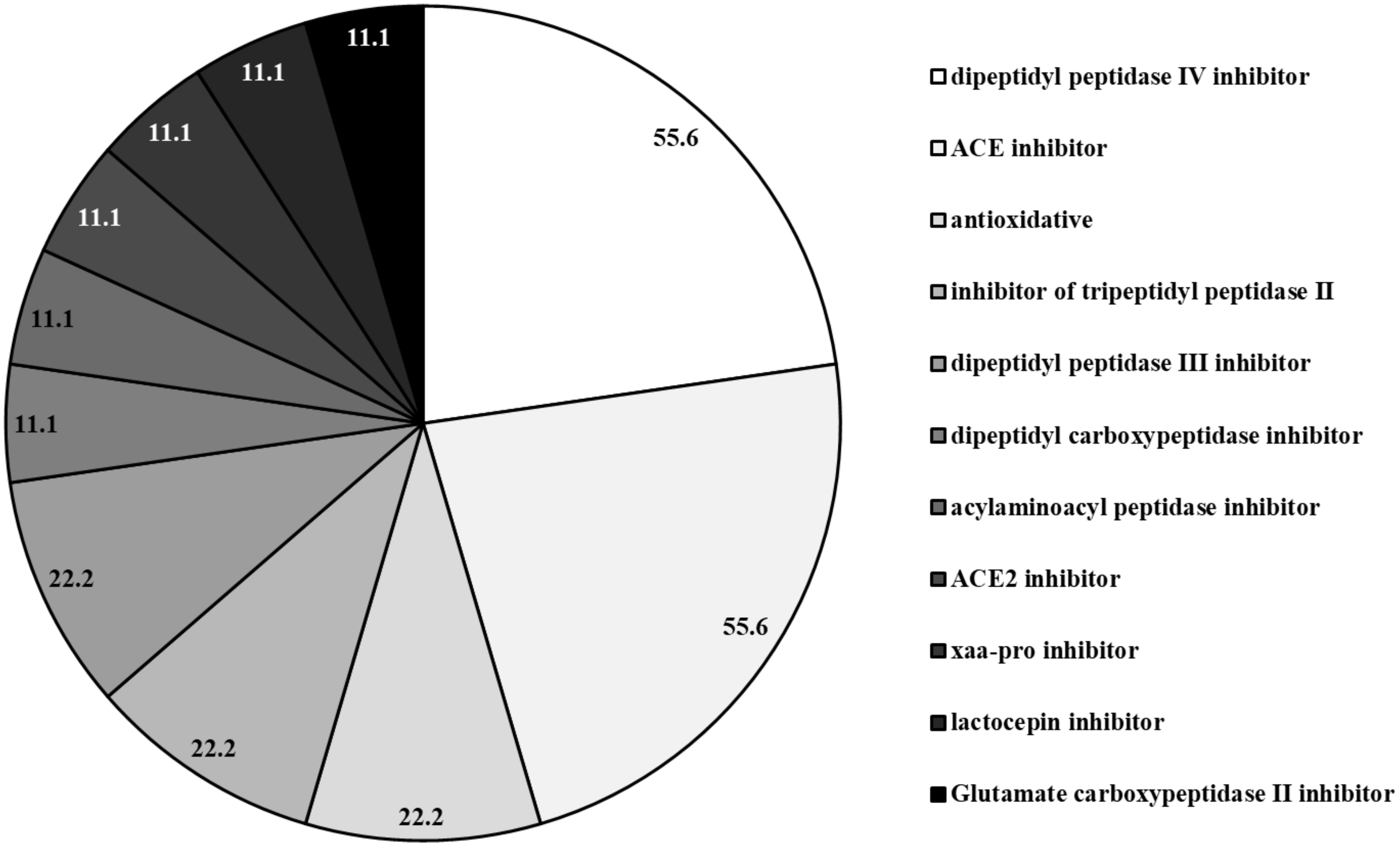

Figure S1. Potential biological activities expressed in percentages of fragments obtained during *in-silico* gastrointestinal digestion with biological activity reported in BIOPEP-UWM database.

Table S2. In-silico evaluation of biological and taste properties of fragments obtained during in-silico gastrointestinal digestion with nonbiological activity reported in BIOPEP-UWM database.

Nº	Peptide	Location	PRK[a]	Toxicity	CPP	FRS score	CHEL score	ACE	DPPIV	Umaminess	Bitterness	Taste BIOPEP
1	DPF	P1, P9, P14, P22	0.95	No	Yes	0.48	0.28	No	Yes	No	No	x
2	DW	P9, P14, P22	0.93	No	Yes	0.47	0.25	Yes	Yes	Yes	Yes	x
3	GDGW	P11, P13	0.93	No	Yes	0.48	0.23	No	Yes	Yes	Yes	x
4	PDPPPL	P16	0.93	No	Yes	0.52	0.32	No	Yes	No	No	x
5	GPSGPPGL	P21	0.93	No	Yes	0.55	0.28	No	Yes	No	Yes	x
6	PPPY	P4	0.92	No	Yes	0.57	0.31	Yes	Yes	No	No	x
7	GPPDPIL	P19, P23	0.91	No	No	0.46	0.27	No	Yes	No	Yes	x
8	PP	P2, P6	0.89	No	Yes	0.50	0.33	No	Yes	No	Yes	x
9	GGGGGGGGGGL	P7, P12	0.89	No	Yes	0.53	0.19	No	Yes	No	Yes	x
10	PPR	P2, P6	0.88	No	Yes	0.48	0.30	No	Yes	No	Yes	x
11	GAGGPGAGGF	P25	0.87	No	No	0.52	0.23	Yes	Yes	No	Yes	x
12	SPSW	P22	0.85	No	Yes	0.44	0.27	No	Yes	No	No	x
13	GAPSF	P10	0.84	No	No	0.42	0.26	No	No	No	No	x
14	GGGL	P7, P12	0.83	No	Yes	0.45	0.25	No	Yes	No	Yes	Bitter
15	GGAPSF	P15, P18	0.80	No	No	0.43	0.25	No	Yes	No	No	x
16	GDSW	P8, P17	0.78	No	Yes	0.41	0.23	No	No	No	No	x
17	GSPL	P18	0.75	No	Yes	0.43	0.28	No	Yes	Yes	No	x
18	TPIF	P24	0.74	No	Yes	0.38	0.25	No	Yes	No	No	x
19	SPPVL	P16	0.67	No	Yes	0.43	0.27	No	Yes	No	No	x
20	GPGS	P13	0.60	No	Yes	0.44	0.27	No	Yes	No	Yes	x
21	GPGSR	P11	0.60	No	Yes	0.42	0.26	No	No	No	Yes	x
22	PA	P14	0.53	No	Yes	0.43	0.30	No	Yes	No	Yes	x
23	GN	P12, P24	0.47	No	Yes	0.42	0.28	No	Yes	Yes	No	x
24	PAH	P22	0.43	No	Yes	0.51	0.32	No	Yes	Yes	No	x
25	ADH	P3	0.18	No	Yes	0.44	0.27	No	Yes	Yes	No	x
26	TQR	P5,P20	0.09	No	Yes	0.38	0.25	No	Yes	No	No	x

[a] Potential biological properties and physicochemical properties were evaluated using the following tools: bioactivity rank from PeptideRanker (PRK) (http://distilldeep.ucd.ie/PeptideRanker/), toxicity from ToxinPred (http://crdd.osdd.net/raghava/toxinpred/), cell-permeating power (CPP) from MLCPP (http://www.thegleelab.org/MLCPP/MLCPP.html), free radical scavening (FRS) and chelating (CHEL) scores from AnOxPePred - 1.0 (https://services.healthtech.dtu.dk/services/AnOxPePred-1.0/). Angiotensin Converting Enzyme (ACE) Inhibition potential from Deepstack-ACE

(https://pmlabqsar.pythonanywhere.com/predict_DeepstackACE) and Dipeptidyl peptidase IV inhibition potential from StackDPPIV (https://pmlabstack.pythonanywhere.com/StackDPPIV). Umami taste were evaluated using the tool UMPred-FRL (https://pmlabstack.pythonanywhere.com/UMPred-FRL) , and bitter taste the tool BERT4Bitter (https://pmlab.pythonanywhere.com/BERT4Bitter). Finally, taste attributes were extracted from BIOPEP-UWM database (https://biochemia.uwm.edu.pl/biopep/start_biopep.php).
